# LGI2 Truncation Causes a Remitting Focal Epilepsy in Dogs

**DOI:** 10.1371/journal.pgen.1002194

**Published:** 2011-07-28

**Authors:** Eija H. Seppälä, Tarja S. Jokinen, Masaki Fukata, Yuko Fukata, Matthew T. Webster, Elinor K. Karlsson, Sami K. Kilpinen, Frank Steffen, Elisabeth Dietschi, Tosso Leeb, Ranja Eklund, Xiaochu Zhao, Jennifer J. Rilstone, Kerstin Lindblad-Toh, Berge A. Minassian, Hannes Lohi

**Affiliations:** 1 Department of Veterinary Biosciences, Department of Medical Genetics, Research Programs Unit, Molecular Medicine, University of Helsinki, Helsinki, Finland; 2 Department of Molecular Genetics, Folkhälsan Research Center, Helsinki, Finland; 3 Department of Clinical Veterinary Sciences, University of Helsinki, Helsinki, Finland; 4 Division of Membrane Physiology, Department of Cell Physiology, National Institute for Physiological Sciences and National Institutes of Natural Sciences, Okazaki, Japan; 5 PRESTO, Japan Science and Technology Agency, Tokyo, Japan; 6 Science for Life Laboratory, Department of Medical Biochemistry and Microbiology, Uppsala University, Uppsala, Sweden; 7 Broad Institute of Harvard and MIT, Cambridge, Massachusetts, United States of America; 8 FAS Center for Systems Biology, Harvard University, Cambridge, Massachusetts, United States of America; 9 Institute for Molecular Medicine Finland (FIMM), University of Helsinki, Helsinki, Finland; 10 Department for Small Animals, Neurology Services, University of Zurich, Zurich, Switzerland; 11 Institute of Genetics, Vetsuisse Faculty, University of Bern, Bern, Switzerland; 12 Department of Clinical Veterinary Medicine, Division of Small Animal Orthopedics and Surgery, Vetsuisse Faculty, University of Bern, Bern, Switzerland; 13 Program in Genetics and Genome Biology, The Hospital for Sick Children, Toronto, Canada; 14 Division of Neurology, Department of Paediatrics, The Hospital for Sick Children, Toronto, Canada; University of Washington, United States of America

## Abstract

One quadrillion synapses are laid in the first two years of postnatal construction of the human brain, which are then pruned until age 10 to 500 trillion synapses composing the final network. Genetic epilepsies are the most common neurological diseases with onset during pruning, affecting 0.5% of 2–10-year-old children, and these epilepsies are often characterized by spontaneous remission. We previously described a remitting epilepsy in the *Lagotto romagnolo* canine breed. Here, we identify the gene defect and affected neurochemical pathway. We reconstructed a large *Lagotto* pedigree of around 34 affected animals. Using genome-wide association in 11 discordant sib-pairs from this pedigree, we mapped the disease locus to a 1.7 Mb region of homozygosity in chromosome 3 where we identified a protein-truncating mutation in the *Lgi2* gene, a homologue of the human epilepsy gene *LGI1*. We show that LGI2, like LGI1, is neuronally secreted and acts on metalloproteinase-lacking members of the ADAM family of neuronal receptors, which function in synapse remodeling, and that LGI2 truncation, like LGI1 truncations, prevents secretion and ADAM interaction. The resulting epilepsy onsets at around seven weeks (equivalent to human two years), and remits by four months (human eight years), versus onset after age eight in the majority of human patients with LGI1 mutations. Finally, we show that *Lgi2* is expressed highly in the immediate post-natal period until halfway through pruning, unlike *Lgi1*, which is expressed in the latter part of pruning and beyond. LGI2 acts at least in part through the same ADAM receptors as LGI1, but earlier, ensuring electrical stability (absence of epilepsy) during pruning years, preceding this same function performed by LGI1 in later years. *LGI2* should be considered a candidate gene for common remitting childhood epilepsies, and LGI2-to-LGI1 transition for mechanisms of childhood epilepsy remission.

## Author Summary

Major remodeling of the neuronal synaptic network occurs during childhood. The quadrillion synapses formed till the end of age two are trimmed to 500 trillion by age 10 through a selective process of strengthening of ideal connections, removal of redundant ones, and formation of new contacts. Very little is known about the basic mechanisms that direct this massive reorganization that leads to the adult brain. The most common epilepsies of humans occur in childhood and are characterized by remission prior to adulthood. Not much is known about their genetics and basic remission mechanisms. We describe here a canine equivalent disease and identify the defective gene, Lgi2. We show that the gene product is a secreted protein and interacts with neuronal ADAM receptors known to be involved in the regulation of synaptic remodeling in the developing brain. Our work sheds important light on the basic mechanisms of the most common neurological disease of children and discloses processes of epilepsy remission. The identification of the first focal epilepsy gene in dogs has also enabled the development of a genetic test to identify carriers for breeding purposes.

## Introduction

Postnatal mammalian brain development proceeds in three phases the first of which is construction of the primary neural network (ages zero to two years in humans, zero to one week in mice, and estimated zero to one to two months in dogs). In humans, this phase generates a network of approximately one quadrillion synapses. The second phase, pruning (ages two to 10 years in humans, seven to 17 days in mice, and estimated two to four months in dogs), is chiefly characterized by massive removal of unneeded or otherwise inappropriate synapses, almost half the original synapses. The third and final phase is the remainder of life, during which synapse numbers remain stable [1]–[3].

Epilepsies are by far the most common neurological diseases in children two to 10 years of age, the three most common of which are Rolandic Epilepsy, Panayiotopoulos syndrome, and Childhood Absence Epilepsy (CAE). The first two of these three syndromes are focal-onset epilepsies where seizures start from defined brain regions, while CAE is a generalized epilepsy where seizures appear to start simultaneously from all brain regions. All three syndromes share a remarkable feature of remission after age 10, i.e. after network pruning is complete [4]. All three are genetically complex syndromes, and paucity of gene information has impeded their understanding, including how and why they remit. To date, a few ion channel mutations (e.g. in *GABRG2, CACNA1H*) have been found in CAE, accounting for far less than 1% of patients with this syndrome [5].

While the above three syndromes begin and end during the pruning phase of neurodevelopment in the vast majority of cases, other genetic epilepsies begin near or after the end of this phase, i.e. after age eight in most cases. These include the generalized Juvenile Myoclonic Epilepsy (JME) (to date with mutations in the *EFHC1* or *GABRA1* genes; penetrance ∼50%) [6], [7] and the focal-onset Autosomal Dominant Lateral Temporal Lobe Epilepsy (ADLTE) (also called Autosomal Dominant Partial Epilepsy with Auditory Features) with mutations in the *LGI1* gene [8] (penetrance 67%) [9]. JME is generally a non-remitting lifelong epilepsy [10]. Remission rate in ADLTE has not been determined, although the literature indicates that most cases remain on seizure medications, unlike Rolandic epilepsy, e.g., where the vast majority do not [10], [11].

In the present work, we show that mutation of the *Lgi2* gene, a gene closely related to *Lgi1*, causes remitting focal-onset epilepsy in dogs between ages one and four months, which is equivalent to human two to eight years. LGI2 belongs to a family of four closely related neuronal proteins including the well-studied LGI1. We report functional and expression studies of LGI2, which, combined with previous LGI1 studies, suggest a novel concept of the basis of remission common in childhood epilepsy.

## Results

### Focal-onset epilepsy in the *Lagotto Romagnolo* canine breed is associated with a truncating mutation of the *Lgi2* gene

The *Lagotto Romagnolo* is an ancient curly-haired water dog (water dogs, or water spaniels, originally served to retrieve game falling in water), which was selected in Italy to become an excellent truffle hunter. The popularity of the breed fluctuated with the truffle industry and in the early 1970s underwent a strong genetic bottleneck to near extinction, when a group of dog lovers decided to save it. The breed has since gained popularity for reasons unrelated to truffle or water hunting, and its numbers are in the thousands spread across most developed countries (http://www.lagottoromagnolo.org/).

The breed is affected by an epilepsy, Benign Familial Juvenile Epilepsy (BFJE), described in detail in reference [12]. Onset is at five to nine weeks of age, and the epilepsy invariably completely remits by four months of age. Remission is so reliable that the epilepsy is considered by many breeders as an unfortunate particularity of the breed and often disregarded. The seizures consist of whole-body tremors sometimes associated with alteration of consciousness. Electroencephalography (EEG) reveals unilateral epileptic discharges in central-parietal and occipital lobes, and magnetic resonance imaging (MRI) is normal. During the months with epilepsy the animals are often ataxic, but this resolves completely as the seizures disappear [12].

**Figure 1:**
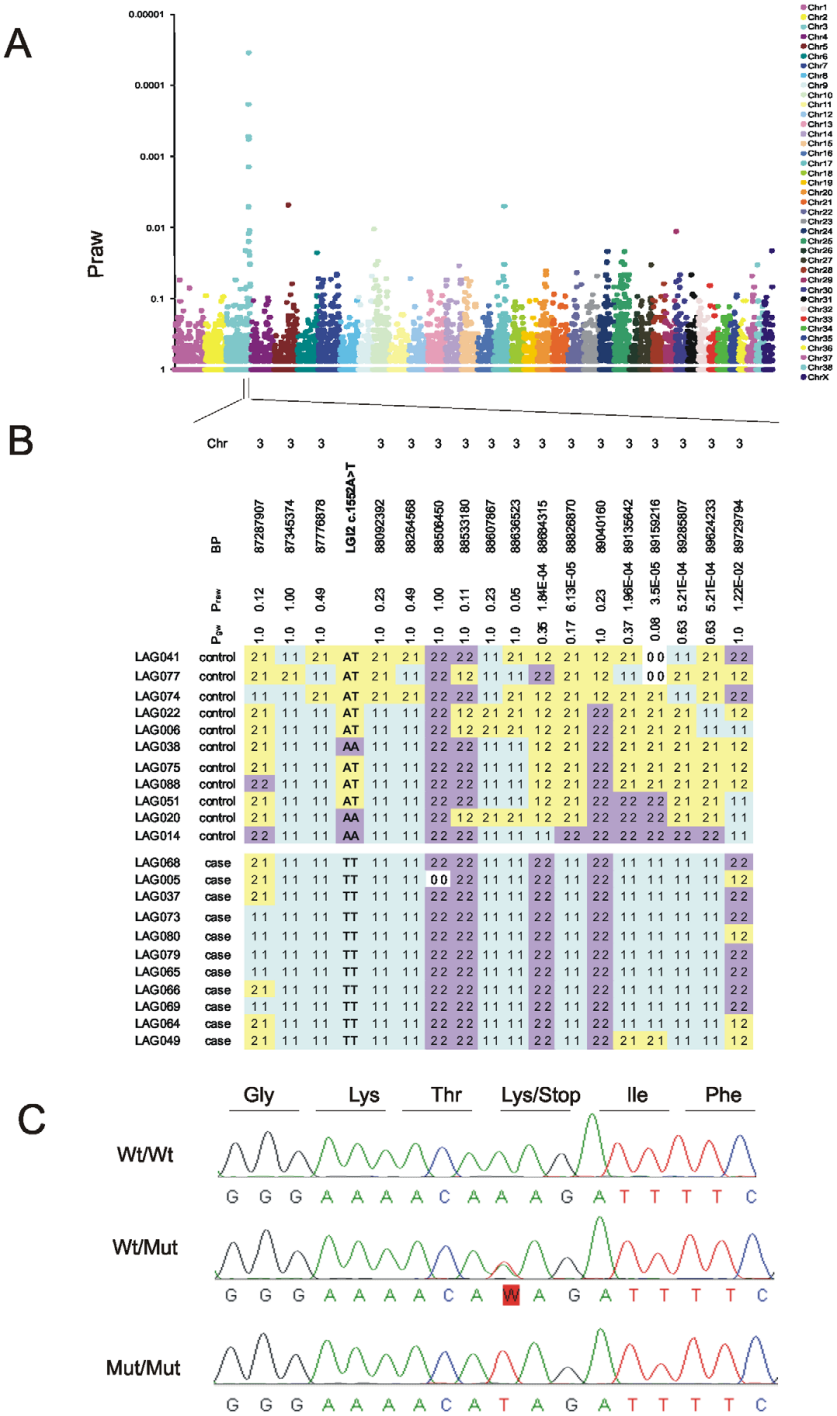
Mapping and identification of the benign focal juvenile epilepsy mutation in *Lagotto Romagnolo*. A) Genome-wide association analysis maps the disease locus to CFA3 with the strongest association at a SNP at position 89,159,216 (P_raw_  = 0.000035 and P_genome-wide_  = 0.08). B) A 1.7 Mb homozygous block spanning from 87.3 Mb to 89.0 Mb is present in affected dogs (bottom set of 11 dogs) and not in unaffected dogs (top set of 11 dogs). C) Sequencing of the *Lgi2* coding regions revealed a homozygous c.1552A>T mutation that causes a premature stop codon in exon 8, resulting in truncation of the last 12 amino acids in the affected cases.

Towards the goal of mapping and identifying the BFJE gene we first reconstructed a large multinational *Lagotto* pedigree from which an example with 212 Finnish dogs including 34 cases is shown in Figure S1. The dogs live in homes as private pets, often in different countries in Europe, or were still with their breeders. Disease segregation suggested autosomal recessive inheritance (Figure S1). Next, we performed a single nucleotide polymorphism (SNP) genome-wide association study with DNA from 11 of the affected dogs and 11 unaffected littermates (discordant sib-pairs) (Figure S1) and found very strong association in a region of chromosome 3 (CFA3), peaking at the marker at base-pair 89159216 (P_raw_ 0.000035; P_genomewide_ 0.08) (Figure 1A and 1B). There was no significant association at any other genomic locus, the next best association being over 100-fold less significant (Figure 1A). Genotype analysis around the 89159216 SNP revealed a 1.7 Mb block of homozygous SNPs between markers at 87.3 Mb and 89.0 Mb in the 11 cases and none of the controls (Figure 1B). This region contains nine genes, including *Lgi2*. Sequencing *Lgi2* revealed an exonic homozygous protein-truncating sequence change, c.1552A>T (p.K518X), in all 11 affected and none of the 11 unaffected animals (Figure 1C). Genotyping a cohort of 140 dogs for the 89159216 SNP, for the *Lgi2* c.1552 sequence change, and for three additional SNPs from the homozygous region revealed extremely high associations including P_raw_ 4.47×10^−16^ at 89159216 and P_raw_ 1.05×10^−23^ (the highest association) at *Lgi2* c.1552 (Table 1). These results strongly suggested that *Lgi2* c.1552A>T (p.K518X) is the BFJE mutation.

**Table 1:** Four highly-associated SNPs spread across the linked 1.7 Mb homozygosity region, together with the c.1552A>T mutation, were genotyped and tested for association from 28 BFJE cases and 112 healthy controls.

Marker	Position	OR	95% CI	P
BICF2G630354291	87328476	76.5	15.1-1870.5	4.47E-13
Lgi2 A1552T	88033499	532.1	95.0-5747.1	1.05E-23
BICF2S23524395	88092392	5.1	0.97-125.3	0.07
BICF2G630355625	88826870	66.9	17.6-476.8	8.14E-15
BICF2G630355982	89135642	68.6	20.9-290.2	4.47E-16

The strongest association is with c.1552A>T mutation confirming that Lgi2 is the causative gene in the region.

Next we studied segregation of the sequence change in the pedigree. Of the 28 affected dogs from which we had samples, 26 (93%) were homozygous *Lgi2* c.1552T (p.518X) (i.e. homozygous for the nonsense codon), two were heterozygous (7%), and none was homozygous for the wild-type (wt) A nucleotide. The two affected dogs that were heterozygous were also heterozygous for the 13 SNP haplotype around the *Lgi2* locus, and we found no evidence for compound heterozygosity as all other variants in the gene were synonymous (Table S1). These results suggested that if the *Lgi2* c.1552A>T (p.K518X) change is the BFJE mutation, it can, in a minority of cases, cause the epilepsy heterozygously. To explore this further, we screened an independent set of 36 sporadic *Lagottos* and found three homozygous for c.1552T, 14 heterozygous (39%), and 19 wild-type (wt). All three dogs homozygous for c.1552T had the syndrome, as did one of the carriers (7%), appearing to confirm the 7% rate of disease through heterozygosity, assuming that *Lgi2* c.1552A>T (p.K518X) is causative.

Among 112 unaffecteds of the genotyped 140 dogs, 69 were homozygous for the wt A nucleotide, 41 were heterozygous, and two, 1.8%, were homozygous for c.1552T (OR = 532, 95%CI: 95.0-5747.1 and p = 1.05×10^−23^). The latter two may be mis-specified as unaffected - clinical information on many of the dogs in the pedigree was obtained through retrospective questionnaires, and it is possible that a breeder missed seizures, as the epilepsy in some cases is mild and short-lived [2]. Alternatively, these two cases may represent incomplete penetrance, assuming, again, that the sequence change we identified is causative. Similarly, other recent recessive gene discoveries indicate incomplete penetrance including canine lens luxation [13], degenerative myoelopathy [14] and a form of neuronal ceroid lipofuscinosis [15].

At this point, there were two possibilities. Either *Lgi2* c.1552A>T (p.K518X) is the BFJE mutation with an incomplete penetrance in a minority of cases, or it is not the causative variant. To gather more data we proceeded with functional studies of the consequences of the truncating sequence change on the LGI2 protein.

### *Lgi2* c.1552A>T (p.K518X) truncation prevents LGI2 secretion and action on neuronal ADAM receptors

**Figure 2:**
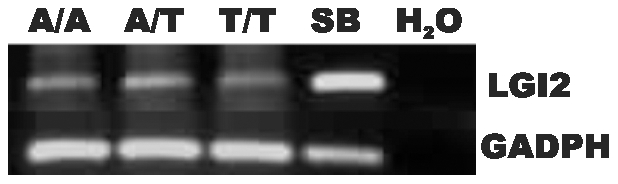
Expression of the mutant *Lgi2* transcript is normal. Total RNA extracted from blood of a healthy (c.1552A/A), a carrier (c.1552A/T), and an affected (c.1552T/T) *Lagotto Romagnolo*, as well as from the cerebellum of a healthy (1552A/A) *Saluki* (referred as SB) was transcribed into cDNAs for amplification by PCR with *Lgi2* exon-specific primers. The truncation mutation does not alter expression level of *Lgi2*.

We first determined whether the c.1552A>T sequence change prevents *Lgi2* mRNA expression, e.g. through mRNA instability. RT-PCR experiments showed no mRNA reduction (Figure 2).

**Figure 3:**
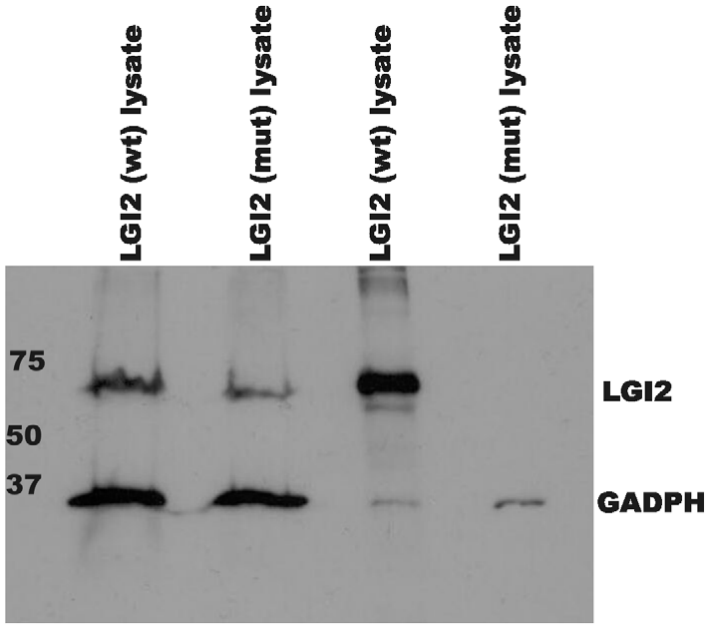
Mutant LGI2 is not secreted in cell cultures. HEK293 cells were transfected with human V5-tagged wt and p.K534X mutant LGI2 clones. Aliquots of lysed cells (lanes 1&2) and culture media (lanes 3&4) were harvested, concentrated, and analyzed with anti-V5 and anti-GADPH antibodies to follow expression of the recombinants. Only wild-type LGI2 protein is found in culture media indicating that the K534X mutation prevents secretion.

LGI2 belongs to a family of neuronally secreted proteins (LGI1 to LGI4) conserved across mammals and composed of N-terminal leucine-rich repeats (LRR) and C-termini containing seven EPTP repeats [3]. K518X truncates LGI2 within the seventh EPTP repeat (exon 8 of the gene) (Figure S2). Similar mutations truncating LGI1 in the EPTP repeats in humans, including in the seventh repeat, cause ADLTE, the human epilepsy with most commonly onset after age eight and persistence through adulthood. Where studied, the vast majority of LGI1 mutations, truncating or otherwise, prevent secretion of the protein encoded by the mutant allele, and ADLTE is therefore usually a disease due to lack of neuronal secretion of half the required amount of LGI [11], [16], [17]. We asked whether the LGI2 K518X truncation prevents LGI2 secretion. We performed western blot experiments with V5-tagged wt and mutant *LGI2* transfected in HEK293 cells and found that while both proteins were present in cell lysates only wt LGI2 was found in the culture medium (Figure 3), indicating that the truncation prevents secretion.

**Figure 4:**
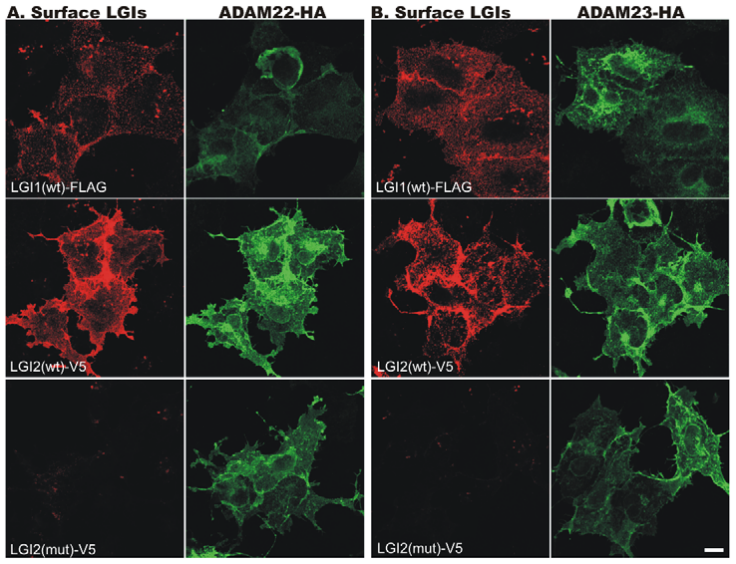
Wild-type (wt) LGI2, like LGI1, binds to ADAM22 and ADAM23 on the cell surface. Indicated cDNAs were co-transfected into COS7 cells, and after 24 hours surface-bound LGI1-Flag and LGI2-V5 (red) were labeled before cell permeabilization and staining of the HA-tagged ADAM22 (A) and ADAM23 (B) proteins (green). Wt Lgi1 and LGI2 bound to both cell surface ADAM receptors, whereas mutant LGI2 was not secreted and did not bind the receptors.

**Figure 5:**
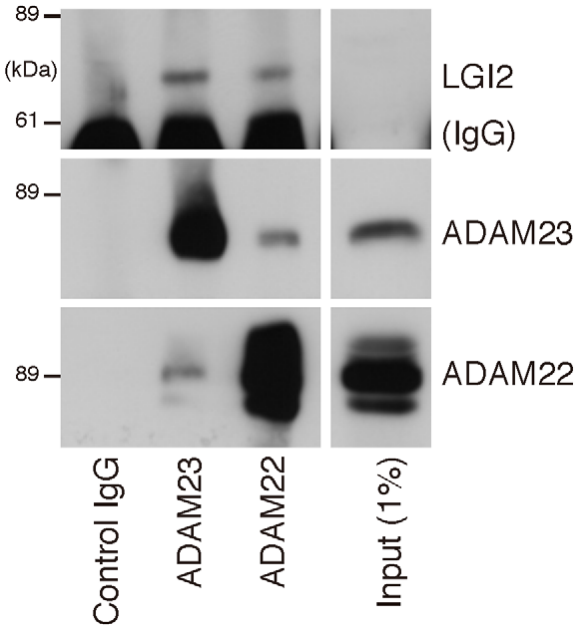
LGI2 interacts with ADAM22 and ADAM23 in rat brain. Western blotting shows that antibodies against ADAM22 or ADAM23 co-precipitate Lgi2 in brain lysates.

Following secretion, LGI1 interacts with a subfamily of the ADAM (a-disintegrin-and-metalloproteinase) family of neuronal membrane proteins [18], [19]. Members of this subfamily, ADAM11, ADAM22 (post-synaptic), and ADAM23 (pre-synaptic), lack the metalloproteinase domain that other ADAMs use to convey extracellular signals intracellularly [19]. To determine whether LGI2 also binds ADAM22, ADAM23 and ADAM11 following secretion, we performed immunofluorescent cell surface-binding assays [18] in permeabilized and non-permeabilized cells by co-expressing wt or truncated LGI2 with different ADAMs. Wt LGI2 was secreted and then bound ADAM22, ADAM23 and ADAM11 expressed on the cell surface (ADAM11 result not shown). Truncated LGI2 was not secreted and did not bind the ADAMs (Figure 4A–4B). We also performed co-immunoprecipitation in rat brain and found that both Adam22 and Adam23 antibodies co-precipitated Lgi2 (Figure 5). In summary, wt LGI2 binds the same ADAM substrates of LGI1 following secretion, and the *Lagotto* K518X mutation prevents secretion and ADAM interaction, in the same fashion as the well-characterized truncating *LGI1* epilepsy mutations.

Summarizing the results to this point, the genome-wide association study revealed a highly significant association in the vicinity of *Lgi2*, an extremely strong association (p = 1.05×10^−23^) with the protein-truncating c.1552A>T (p.K518X) sequence change in the gene, and no significant association with any other locus. *Lgi2* is a close homologue of the epilepsy (ADLTE) gene *LGI1*, and the *Lgi2* truncating mutation is closely similar to the most common type of epilepsy-causing mutations in *LGI1*. The consequence of the truncation on Lgi2 is identical to the consequence of truncation on LGI1, prevention of neuronal secretion and binding to ADAM receptors, which is presently the most favored mechanism of epileptogenesis in ADLTE. Finally, *LGI1* mutations, including truncation mutations, are non-penetrant in 33% of individuals, compared to 1.8% non-penetrance in the case of the canine *Lgi2* truncation. Considering all the above, we believe the data meet the burden of proof that *Lgi2* c.1552A>T (p.K518X) is the BFJE mutation. BFJE is transmitted in imperfect Mendelian fashion. In the vast majority of cases, 93%, homozygous mutation is required for the disease to manifest. In a minority, 7%, heterozygosity suffices. Conversely, 1.8% of dogs may be resistant to seizing despite homozygous mutation. Finally, we found no dog with homozygous wt genotype at *Lgi2* c.1552 that has BFJE.

### *Lgi2* is highly expressed during the first phase of postnatal development (neural network construction phase) and diminishes and plateaus during the network pruning phase

**Figure 6:**
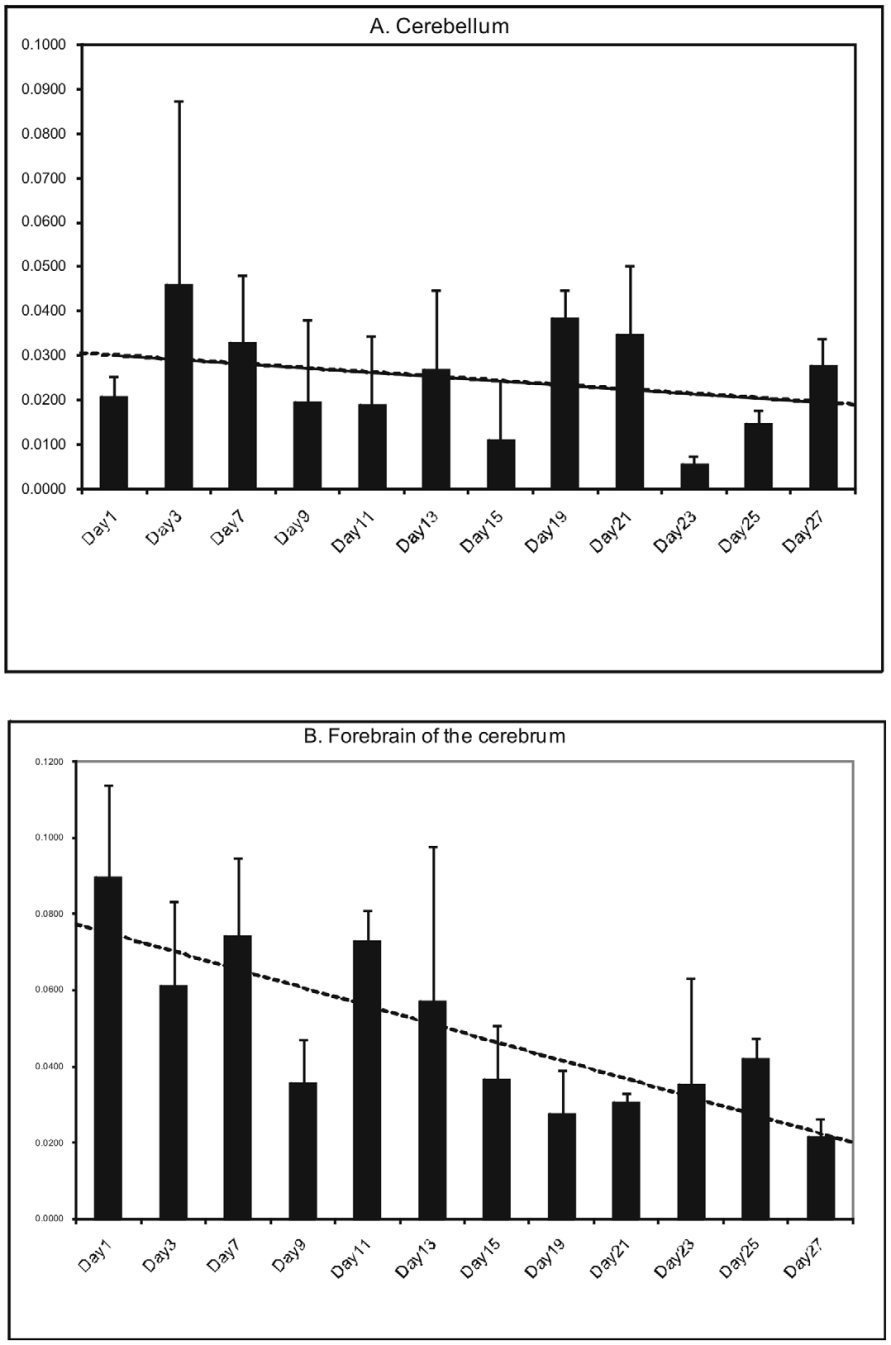
Developmental expression of LGI2 in mouse brain. Mouse cerebelli (A) and forebrains (B) were harvested postnatally every other day for the first four weeks of life (except days 5 and 17) and *Lgi2* transcript levels were measured by quantitative RT-PCR. *Lgi2* expression is at the highest levels in the forebrain at birth but decreases significantly after p13. In the cerebellum Lgi2 levels remain stable. Trendline is shown by dashed lines.

Mouse studies show that LGI1 starts being expressed midway through the synapse pruning phase of brain development (after postnatal day 13 (p13)), and gradually increases to reach high and stable adult levels by the end of this phase (after p17) [20], [21]. Not surprisingly, the mice lacking *LGI1* develop seizures after mid-phase pruning [22] and the great majority of human patients with ADLTE have onset of their epilepsy after age 10 years, the end of the pruning phase in humans, with the remaining few having initial seizures in the latter half of this phase [11], [16], [17]. However, it is worth noting that the very first seizures in ADLTE are often auditory seizures, which might not initially be appreciated to be seizures and might have occurred earlier than what is currently documented in the literature. Because BFJE occurs only in ages equivalent to human two to eight years, we sought to determine whether expression of *LGI2* differs from that of *LGI1*. We examined the expression profile of all four *LGI* genes in adult tissues using the human GeneSapiens expression database [4] and found that the amount of *LGI2* in adult brain is much lower than that of the other three (Figure S3). We next chased *Lgi2* expression levels in mouse forebrain and cerebellum by performing quantitative RT-PCR every other day from birth till 27 days. *Lgi2* expression in the cerebellum did not change appreciably over this time (Figure 6A). Expression in the forebrain, on the other hand, was highest at birth and through phase one of postnatal development (neural network construction phase), and declined to half the original amount by midway through the pruning phase (Figure 6B). Considering that BFJE occurs only during the pruning phase, these results suggest that LGI2's main functions take place in the developmental phase preceding the phase in which the epilepsy occurs.

## Discussion

Epilepsy is a common symptom of insult to the brain from various causes including tumors, trauma, stroke, and neurodegenerative disease. For example, the most common cause of epilepsy in the elderly is stroke and in neonates hypoxic ischemic encephalopathy. However, epilepsy can also be a disease onto itself, where seizures are the only or preponderant neurological symptom, i.e. the brain is normal except for its propensity to seize. Resolving the basic mechanisms of this type of ‘pure’ epilepsy is expected to provide the clearest insights into epileptogenesis. As mentioned, these pure epilepsy syndromes (sometimes called idiopathic, Greek for ‘disease onto itself’) are the commonest neurological diseases with onset in two to 10 year-old children, and in this age group most are genetic, commonly polygenic, and often characterized by remission in adolescence [5].

Genetic-idiopathic epilepsy syndromes are the most common neurological diseases of dogs, in some breeds 10 times more common than in humans [23]. In the present work we identify the first of the canine idiopathic epilepsy genes, in a remitting syndrome with onset and offset equivalent to human childhood two to 10 years. This epilepsy can now be eliminated from the *Lagotto* through selective breeding. Carrier frequency of the mutation is very high. We tested 576 *Lagottos* from three different countries and found a carrier rate of 32% (Table S2). On the other hand, the mutation appears restricted to this particular epilepsy in this particular breed. We tested 121 epileptic dogs from 40 different breeds, including Barbets, a *Lagotto*-related French water spaniel breed afflicted with a separate epilepsy, and none carries the BFJE mutation (Table S3).

Genetic epilepsies of various types, as simple or complex traits, are highly enriched in various canine breeds, including Miniature Wirehaired Dacshunds [24], Finnish Spitzs [25] and Belgian Shepherds [23] due to pure-breeding. Each of these traits is in genetic isolation within its corresponding breed. This vastly improves signal to noise ratio in genetic studies compared to human populations [26], which should facilitate mapping epilepsy genes. The *Lagotto* themselves segregate a second epilepsy with onset in adulthood completely distinct from BFJE [12]. We have established that this second epilepsy is not associated with the BFJE mutation (Table S1), and are presently mapping its gene(s). Five out of the six adult-onset cases with persistent seizures in our pedigree were genotyped and only one of them was homozygous for the BFJE mutation. However, the puppyhood history of this case is unknown and it was impossible to confirm retrospectively whether this case has also had BFJE. This case has an affected littermate with classical BFJE who is homozygous for the mutation. On the other hand, all the other genotyped adult-onset dogs were wildtypes strongly suggesting that this form of epilepsy has its own genetic cause, and that this single homozygous case may have suffered from both BFJE and the adult-onset form of epilepsy.

The BFJE gene is a homolog of the human epilepsy gene *LGI1*. LGI1 is neuronally secreted and binds three metalloproteinase-lacking ADAM receptors. Significant progress has started to be made in elucidating LGI1's functions at these receptors. LGI1 interaction with post-synaptic ADAM22 strengthens and stabilizes ADAM22-containing synapses [18], [21], [22]. Interaction with pre-synaptic ADAM23 enhances neurite outgrowth from ADAM23-containing axons [27]. Through its seven-bladed β-propeller structure (encoded by the EPTP repeats), LGI1 simultaneously binds ADAM23 and ADAM22, pulling pre and post-synaptic membranes together, physically stabilizing synapses containing these two proteins and strengthening neurotransmission in these synapses [22]. Importantly, LGI1 regulates neuronal terminal pruning and maturation, again through a combined pre and post-synaptic action [21].

Humans not expressing or secreting LGI1 from one allele develop epilepsy starting in the vast majority of cases after age eight and seeming to persist in adulthood in many cases. Mice completely lacking LGI1 are normal until midway through the pruning phase of brain development (∼P13), when LGI1 would normally have started being expressed, after which they develop seizures that progressively worsen as LGI1's amounts would normally have progressively increased, and die of violent convulsions by four weeks of life [22], [28], [29]. These results show that LGI1 is a vital protein, vital specifically in protecting the brain against seizures. In humans its partial loss results in epilepsy, and only epilepsy, and in the mice its complete loss leads to death from epilepsy prior to the presence of any other neurological symptom. This vital anti-epileptic role is mediated at least in part through the above three ADAM receptors suggesting that the LGI1-ADAM complexes and their related pathways are essential components of neural network electrical stability in the maturing and mature brain.

We show in our study that lack of secretion of LGI2 is also associated with epilepsy, at an earlier stage of development, and secreted LGI2 interacts with the same ADAM receptors as LGI1, suggesting that LGI2 participates in protecting the brain against seizures during the pruning phase of neurodevelopment at least in part through the same system utilized by LGI1 in the subsequent phase. Importantly, LGI2 expression is highest in the phase preceding pruning and epilepsy. This suggests that the LGI2 anti-epileptic activity anticipates the pruning phase, i.e. LGI2 acts during the network construction phase to help prepare a network that will not seize during the pruning phase. To date, there has been no compelling evidence-based theory of why so many epilepsies of childhood begin and end with the start and end of the pruning phase. Our results, combined with the body of LGI1 work, suggest the following. Construction of the initial network includes mechanisms, in which LGI2 participates, that ensure that the network will not seize during the pruning phase. Defects in these anticipatory anti-epileptic processes result in epilepsy as the massive changes of the pruning phase commence. The pruning phase itself encompasses mechanisms, in which LGI1 participates, that ensure that the pruned and remodeled network to serve the rest of the animal's life is electrically stable. These mechanisms are able to correct or compensate for earlier instabilities, e.g. those introduced by LGI2 deficiency, resulting in the remission that characterizes so many childhood idiopathic epilepsies.

Of the remaining two LGI proteins, LGI4 appears not to have a major role in the central nervous system. Instead, its chief function appears to be in regulating neuron-Schwann cell interaction, its secretion defect resulting in inability of Schwann cells to correctly myelinate peripheral nerves, resulting in peripheral nervous system hypomyelination and the murine claw-paw phenotype [30]. LGI3's function, on the other hand, appears to be similar to that of LGI1, as there is evidence that like LGI1 it regulates neurite outgrowth [31]. LGI3 starts being expressed at p7 in mouse, i.e. at the very start of the pruning phase, has steady and high expression throughout the brain in adulthood, and interacts with ADAM22 and ADAM23, as well as presynaptic SNARE complexes [31]–[34]. However, LGI3 does not rescue the LGI1 knockout mouse epilepsy [22], and therefore the two proteins are at least not interchangeable.

Four phenotypes have been associated with LGI1. The first is normalcy in the up to 33% of patients with heterozygous mutations in ADLTE families. Second is ADLTE in the remaining patients with heterozygous *LGI1* mutations. The third is the recent realization that the acquired autoimmune epilepsy syndrome Limbic Encephalitis, long thought to be due to auto-antibodies against a potassium channel, is in fact due to an auto-antibody against LGI1. As expected, all patients with this condition are over 10 years of age [35]. The final phenotype is the intractable and fatal mid-pruning phase-onset murine epilepsy caused by complete LGI1 deficiency. In the clinic, we not infrequently encounter previously normal children with onset of explosive catastrophic epilepsy. Homozygous mutations in *LGI1* (and possibly *LGI3*) should be considered as a possible cause of this presentation.

The phenotype associated with LGI2 in the present study is of a remitting epilepsy with focal onset and centrotemporal and occipital spikes on EEG, occurring within the age range equivalent to human two to 10 years. Two of the most common human epilepsy syndromes occur in this age range, Rolandic Epilepsy, which is focal in onset with centrotemporal spikes on EEG, and Panayiotopoulos Syndrome, again focal-onset, with both centrotemporal and occipital spikes on EEG. *LGI2* should be considered a candidate gene in these common epilepsies.

## Materials and Methods

### Ethics statement

We have collected blood samples from privately owned pets for our genetic studies and have a valid ethical permission for the proposed blood sampling in the study, ESLH-2009-07827/Ym-23 (Oct 2009–Oct 2012) from the Animal Ethic Committee, The State Provincial Office of Southern Finland, P.O. B150, 13101 Hämeenlinna.

### Study population

Mapping of the benign focal juvenile epilepsy (BFJE) locus in *Lagotto Romagnolo* was based on clinically studied litters from Finland [12], German and Switzerland including 25 epileptic puppies, 17 healthy littermates and 12 parents. Furthermore, based on the retrospective questionnaire-based phenotype information, we expanded our study cohort to a total of 112 healthy LR dogs and 28 BFJE cases and collected also 36 sporadic dogs. Additionally, the study population included also five adult-onset epilepsy LR cases [12] and our clinically diagnosed juvenile epilepsy cases from other breeds including Barbets, Collies and German Shepherds (Table S1). Population-based allele and genotype frequencies were estimated from a population of 576 *Lagotto* samples from three different countries (Table S2). EDTA-blood samples were collected and genomic DNA was extracted using a commercially available kit (Puregene, Gentra Systems, Minneapolis, MN). The Finnish Kennel Club's breeding database, Koiranet, was utilized for pedigrees.

### Genome-wide association study (GWAS)

Altogether 22 dogs including seven discordant full sibs and four half-sibs were selected for GWAS. Genotyping was performed with Affymetrix's Canine SNP Array version 1 containing 26,578 markers (Affymetrix, Santa Clara, CA). The SNP association analysis was performed with PLINK software [36] with the criteria of MAF <0.05, call rate >75% and <25% of missing genotypes in individual dogs. After applying these filters, 17,273 SNPs remained in the analysis for all dogs. Genome-wide significance was ascertained through 10 000 random permutations of epilepsy phenotype.

### Mutation screening

Exons and splice junctions were amplified by PCR with primers listed in . The PCR products were purified with ExoSAP-IT kit (USB Corporation, Cleveland, Ohio) and sequenced with an ABI Prism 3730xl DNA analyzer (Applied Biosystems, Foster City, CA). To confirm that the *Lgi2* is the causative gene in the associated 1.7 Mb region we sequenced four SNPs around the associated homozygozity region together with the mutation in 112 healthy and 28 epileptic *Lagottos*. Odds ratio was calculated using conditional maximum likelihood estimation and corresponding 95% CI was calculated from Fisher exact test. The calculations were done with R statistical software package. Absence of the mutation in other breeds was studied by sequencing epileptic cases from altogether 40 different breeds (Table S3).

### Transcript analyses

To study the effect of the nonsense mutation on the stability of *Lgi2* transcript, total RNA was isolated from an affected and a healthy *Lagotto* dog from peripheral blood using PAXgene Blood RNA Kit (PreAnalytix, Hombrechtikon, Switzerland). Total RNA isolated from the cerebellum of a Saluki puppy euthanized due to hernia diaphragmatica was used as an amplification control. cDNA synthesis was performed using the High Capacity RNA-to-cDNA kit (Applied Biosystems, Foster City, CA), and exon 4- and exon 8-specific primers (Table S4) were used to amplify *Lgi2* by PCR. Transcriptional profiling of the *LGI2* mRNA expression levels across a large number of human tissues was retrieved from the public GeneSapiens (PMID: 18803840) database containing data from a meta-analysis of 9873 samples analyzed using the Affymetrix gene expression microarrays [37].

### Cell culture, transfections, and Western blotting

To study the effect of the mutation on the expression and secretion of LGI2 in cell culture, we obtained the human *LGI2* clone from GeneScript Corporation (Piscataway, NJ). The mutant *LGI2* clone including the premature stop codon (p.K534X corresponding to canine p.K518X) was prepared from the wt clone and both were cloned into the pcDNA3.1D/V5-His vector in frame with the C-terminal V5-tag using pcDNA3.1 Directional TOPO Expression Kit (Invitrogen, Carlsbad, CA). The recombinant constructs were confirmed by sequencing. HEK293 cells were grown in DMEM-GLUTAMAX medium (Gibco Laboratories, North Andover, MA) supplemented with 10% fetal calf serum (FCS), 100 IU/ml penicillin, 100 µg/ml streptomycin and 1 mM Sodium Pyruvate and transiently transfected with the FuGENE 6 reagent (Roche Diagnostics, Indianapolis, IN) according to the manufacturer's instructions. Expression of the wt and mutant *LGI2* were analyzed 48 hours after transfection by immunostaining on Western blots with anti-V5 antibodies from cell lysates and culture media. Media samples were concentrated 100-fold with Sentricon-10k concentrator (Millipore, Billerica, MA) before loading onto gel. GADPH was used as internal loading control using anti-GADPH antibodies. Proteins were visualized using the enhanced chemiluminescence method.

### Cell-surface binding assay

COS7 cells were co-transfected using human V5-tagged wt LGI2B or LGI2B p.K534X mutant or rat FLAG-tagged wt LGI1 with mouse HA-tagged Adam22 or Adam23. LGI1 and ADAM clones were described previously [18]. 24 hours after transfection, cells were fixed with 2% paraformaldehyde at RT for 10 min, blocked with PBS containing 10 mg/ml BSA and stained with anti-Flag or anti-V5 antibodies followed by Cy3-conjugated secondary antibody without permeabilization to visualize only the cell-surface bound LGIs. Then, the cells were permeabilized with 0.1% Triton X-100 for 10 min, blocked with PBS containing 10 mg/ml BSA, and stained with anti-HA polyclonal antibody, followed by Alexa488-conjugated secondary antibody. Fluorescent images were taken with a confocal laser microscopy system (Carl Zeiss LSM 510; Carl Zeis, Oberkochen, Germany).

### Developmental expression

To study the developmental expression of the *Lgi2* transcript a colony of C57/BL6 mouse was established for tissue and RNA harvesting. Every other day after birth (except days 5 and 17) one mouse was sacrificed and the forebrain of the cerebrum and the cerebellum were harvested and deep-frozen in liquid nitrogen before total RNA isolation by the QIAGEN RNeasy mini kit. The isolated RNA was DNase I-treated before RT-PCR by the SuperScript First-Strand Synthesis System (Invitrogen, Carlsbad, CA). Quantitative PCR was performed using a SYBR Green method with MxPro-3005P multiplex Quantitative PCR systems. *Lgi2*-specific forward, atgtgtacgtggccatcgctca, and reverse, caaacttggtccagctctcgtcgta, primers were used for amplification.

## Supporting Information

Figure S1 Segregation of the *LGI2* c.1552A>T mutation in a Finnish *Lagotto Romagnolo* pedigree, which is a part of our larger and more complex multinational pedigree. Squares denote males, circles females. The benign focal juvenile epilepsy (BFJE) cases and adult-onset epilepsy cases are marked with black and red, respectively. Dogs with DNA samples are marked according to the *Lgi2* mutation genotypes (A/A, wild type; A/T, heterozygous carrier; T/T, homozygous for epilepsy mutation). Clinically studied litters are numbered 1-12. Red arrows indicate the two BFJE cases that were carriers of the mutation while all other BFJE cases were homozygous for the mutation. Four out of the five adult-onset cases genotyped were wild-types. The puppyhood history of the only adult-onset case homozygous for the BFJE mutation (T/T) remains unclear, however, the dog differs from the other BFJE cases still having persistent seizures as an adult without remission [12]. It is possible that this case may have had both BFJE and the adult-onset epilepsy.
(TIF)

Figure S2 Alignment of human (NP_060646.2), chimpanzee (XP_526541.1), dog (XP_545971.2), cow (XP_614279.2), mouse (NP_659194.1,LGI2A), rat (XP_223494.4), chicken (XP_001232758.1) and zebra fish (NP_001034730.1) LGI2 protein sequences. The LRR domain is shown in grey, and the EPTP/EAR repeats in yellow. The canine p.K518X mutation site at the end of the protein is indicated by a red arrow.
(RTF)

Figure S3 Expression profiles of the LGI family members. Box plot analysis of the median *LGI1* (A), *LGI2* (B), *LGI3*(C) and *LGI4* (D) expression levels across different normal non-CNS tissues (green) and healthy CNS tissues (red). The number of samples in each category is shown in parentheses. The box refers to the quartile distribution (25–75%) range, with the median shown as a black horizontal line. In addition, the 95% range and individual outlier samples are shown.
(PDF)

Table S1 The coding regions and splice sites were screened for additional variants in the *Lgi2* gene in two heterozygous affecteds (LAG001 and LAG099) and a set of adult-onset epileptic *Lagottos* or other puppies with juvenile epilepsy from other breeds including *Barbets, Collies and German Shepherds*. Although several variants were found none of them appear disease-causing.
(DOC)

Table S2 Genotype frequencies of the *Lgi2* c.1552A>T (p.K518X) mutation tested from *Lagotto Romagnolo* dogs in different countries.
(DOC)

Table S3 The *Lgi2* c.1552A>T mutation is breed-specific. Mutation was screened altogether from 114 adult-onset epilepsy cases and eight juvenile epilepsy cases that represented 40 different breeds. None of the studied dogs carried the mutant allele present in *Lagottos*.
(DOC)

Table S4 Primers used in the study.
(DOC)
