# Supplementary figures and images for: LGI2 Truncation Causes a Remitting Focal Epilepsy in Dogs
Source: PLoS Genet. 2011 Jul 28;7(7):e1002194. doi: 10.1371/journal.pgen.1002194 (PMC3145619; doi:10.1371/journal.pgen.1002194)

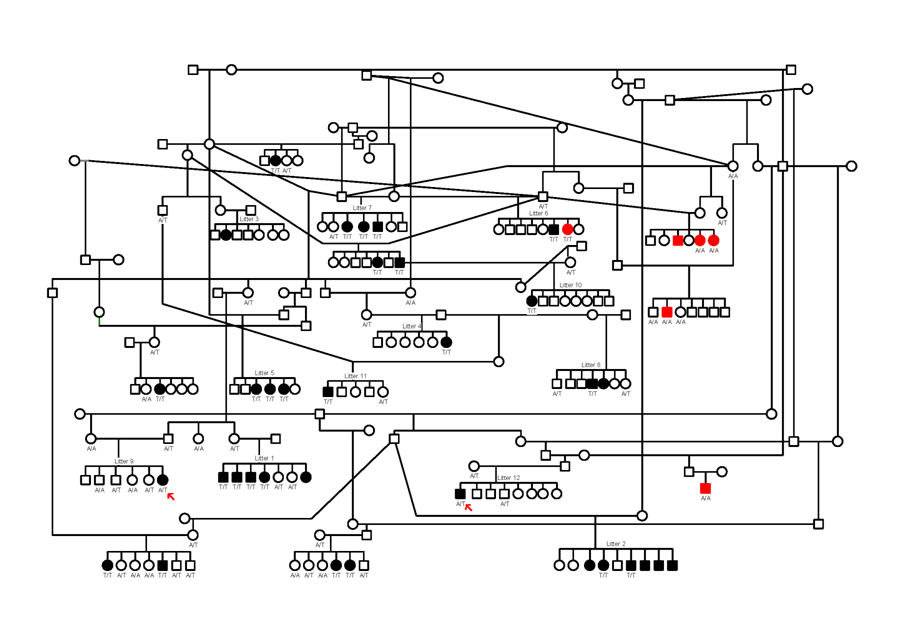

Supplement: Figure S1 — Segregation of the LGI2 c.1552A>T mutation in a Finnish Lagotto Romagnolo pedigree, which is a part of our larger and more complex multinational pedigree. Squares denote males, circles females. The benign focal juvenile epilepsy (BFJE) cases and adult-onset epilepsy cases are marked with black and red, respectively. Dogs with DNA samples are marked according to the Lgi2 mutation genotypes (A/A, wild type; A/T, heterozygous carrier; T/T, homozygous for epilepsy mutation). Clinically studied litters are numbered 1-12. Red arrows indicate the two BFJE cases that were carriers of the mutation while all other BFJE cases were homozygous for the mutation. Four out of the five adult-onset cases genotyped were wild-types. The puppyhood history of the only adult-onset case homozygous for the BFJE mutation (T/T) remains unclear, however, the dog differs from the other BFJE cases still having persistent seizures as an adult without remission [12]. It is possible that this case may have had both BFJE and the adult-onset epilepsy. (TIF) [file pgen.1002194.s001.tif]

**A.**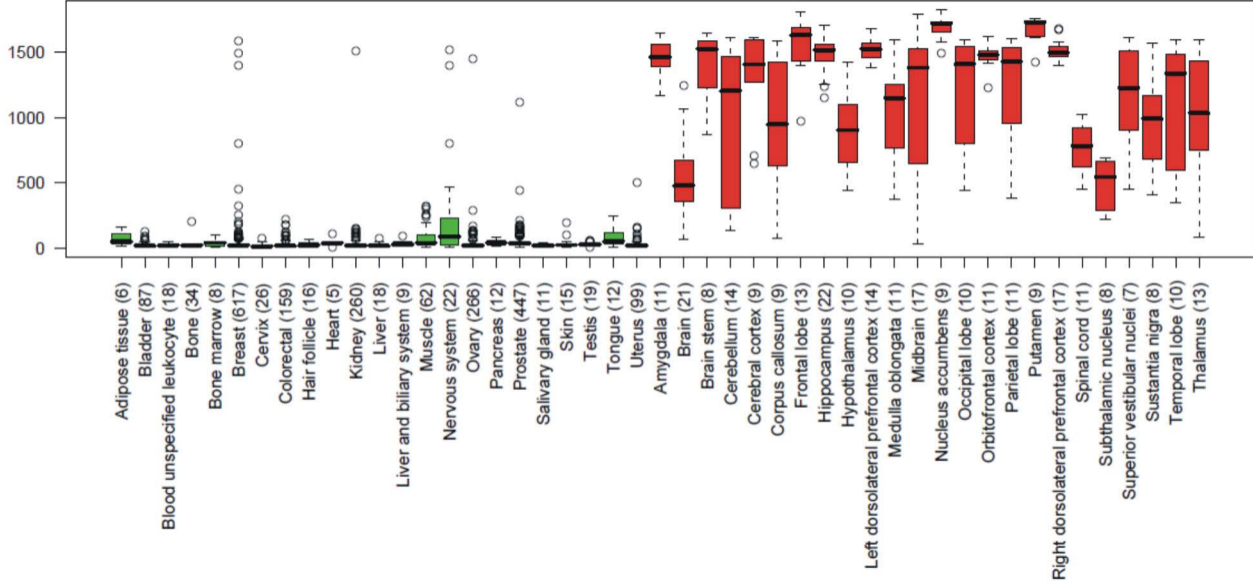

B.

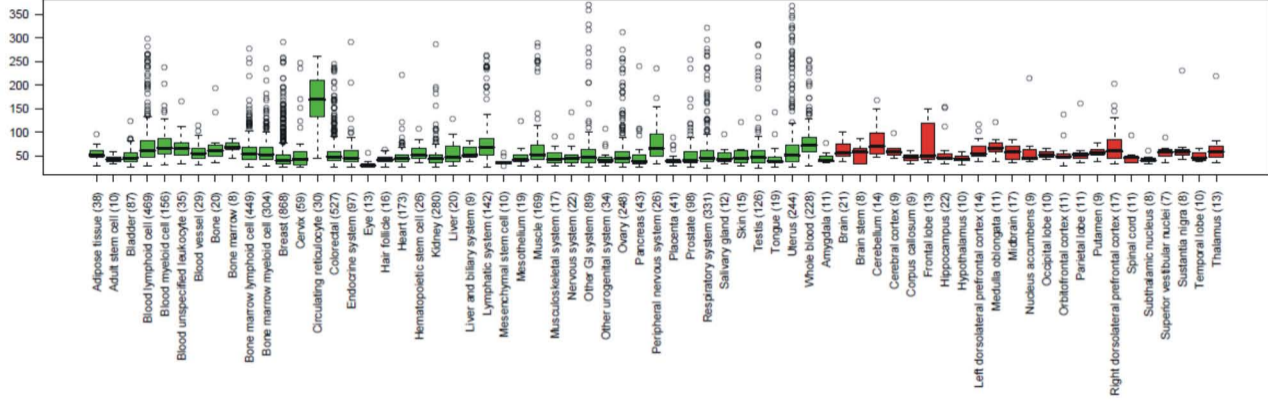

c.

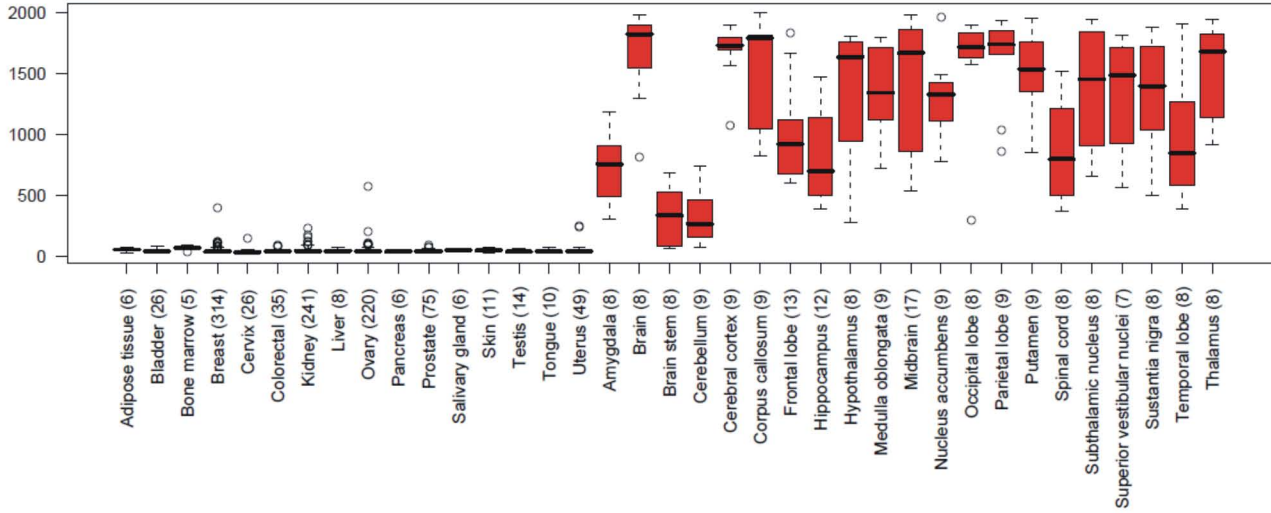

**D.**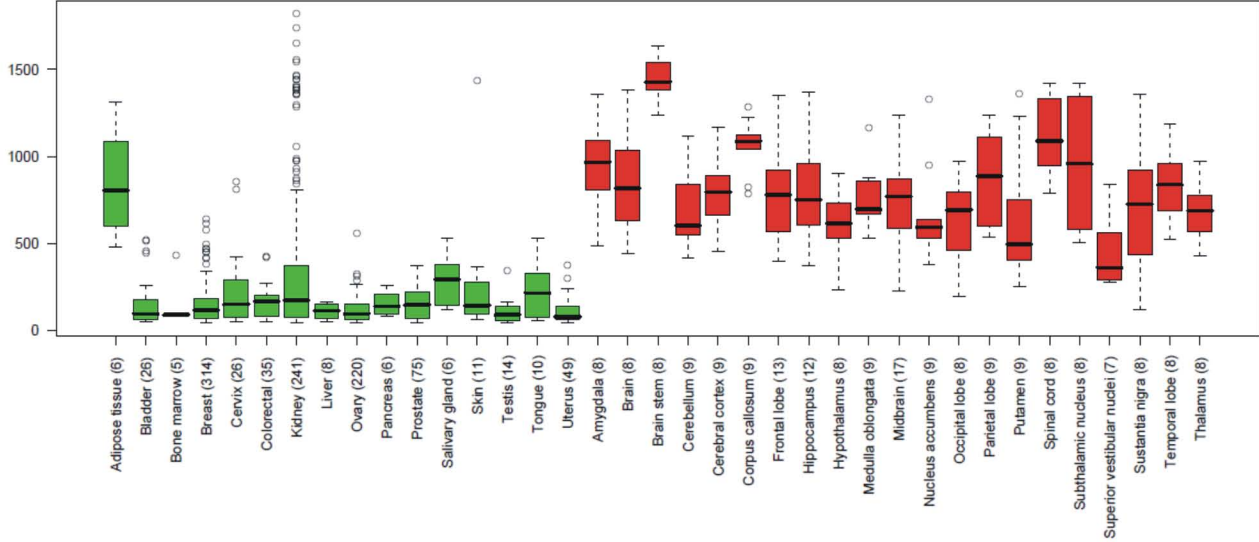

Supplement: Figure S3 — Expression profiles of the LGI family members. Box plot analysis of the median LGI1 (A), LGI2 (B), LGI3(C) and LGI4 (D) expression levels across different normal non-CNS tissues (green) and healthy CNS tissues (red). The number of samples in each category is shown in parentheses. The box refers to the quartile distribution (25–75%) range, with the median shown as a black horizontal line. In addition, the 95% range and individual outlier samples are shown. (PDF) [file pgen.1002194.s003.pdf]
