# Supplementary material for: LGI2 Truncation Causes a Remitting Focal Epilepsy in Dogs
Source: PLoS Genet. 2011 Jul 28;7(7):e1002194. doi: 10.1371/journal.pgen.1002194 (PMC3145619; doi:10.1371/journal.pgen.1002194)
Supplement: Figure S2 — Alignment of human (NP_060646.2), chimpanzee (XP_526541.1), dog (XP_545971.2), cow (XP_614279.2), mouse (NP_659194.1,LGI2A), rat (XP_223494.4), chicken (XP_001232758.1) and zebra fish (NP_001034730.1) LGI2 protein sequences. The LRR domain is shown in grey, and the EPTP/EAR repeats in yellow. The canine p.K518X mutation site at the end of the protein is indicated by a red arrow. (RTF) [file pgen.1002194.s002.rtf]

HUMAN                --------------------------------------------------
CHIMPANZEE               --------------------------------------------------
DOG                      --------------------------------------------------
COW                  1   ----------------------------------------MGISGSCWKL   10
MOUSE                    --------------------------------------------------
RAT                  1   MSERGGLMTQALMSLGMQNDLSNLPALTRLPKRHPSSHMPSGYSSACVYL   50
CHICKEN                  --------------------------------------------------
ZEBRA FISH               --------------------------------------------------


HUMAN              	      --------------------------------------------------
CHIMPANZEE               --------------------------------------------------
DOG                      --------------------------------------------------
COW                 11   LRQLPIEVRNEPPKCARCGKFNFCLWTPAARNWPAKGSWEGSRRGRGARR   60
MOUSE                    --------------------------------------------------
RAT                 51   LVSKPRENKPQCYMKKDEDKRGIYDYLLLLYDRQQKVADFSELRCSGDSD   100
CHICKEN                  --------------------------------------------------
ZEBRA FISH               --------------------------------------------------


HUMAN              	      --------------------------------------------------
CHIMPANZEE               --------------------------------------------------
DOG                      --------------------------------------------------
COW                 61   GRCEAPSPRAGARPALFIGQSGLRARVLMSGGRGCGGRSGGGRPAARARR   110
MOUSE                    --------------------------------------------------
RAT                101   LHSDTASAGEAGAAAAQQPGRGWGGAEWGWRPACCSAAARPPTPAAPASS   150
CHICKEN              1   --------------------------------------MAGPLRLLALWA   12
ZEBRA FISH               --------------------------------------------------


HUMAN              	  1   ----------------------------------------MALRRGGCGA   10
CHIMPANZEE           1   ----------------------------------------MALRRGGCGA   10
DOG                  1   ----------------------------------------MNVY----GP   6
COW                111   RRSSEQRPRLQRRRVRARG------AAAGVVAGTGTGDPGMALRRGGGGP   154
MOUSE                1   ----------------------------------------MALWRGG-GA   9
RAT                151   PAALAADRQAAPASAAALGPSARRGAAAAAGVRAGTGGPGMALRRGG-GA   199
CHICKEN             13   VALLLLPPPAAPRRP------------------PPRCAPPCSCWRESALC   44
ZEBRA FISH           1   -------------------------------------------------M   1


HUMAN              	 11   LGLLLLLLGAACLIPRSAQVRRLARCPATCSCTKESIICVGSSWVPRIVP   60
CHIMPANZEE          11   LGLLLLLLGAACLIPRSAQVRRLARCPATCSCTKESIICVGSSWVPRIVP   60
DOG                  7   ------------LVRPGAQVRRLARCSATCSCTKESIICVGSSWVPRTVP   44
COW                155   GLLLLLLLGTACLIPPSAQVRRLVRCPATCSCTKESIICVGSSWVPRIVP   204
MOUSE               10   LG--LLLLSAACLIPPSAQVRRLARCPATCSCTKESIICVGSSWVPRIVP   57
RAT                200   LG--LLLLSAACLIPPSAQVRRLARCPATCSCTKESIICVGSSWVPRIVP   247
CHICKEN             45   MG----AAGAPRSLPAGFGSLPPPRCAPPCSCWRESALCVGAAGAPRSLP   90
ZEBRA FISH           2   RTVVIIWALLLYLAPLGNTAKKAFKCPSSCSCSKESIICVGSSYVPRYIP   51


HUMAN              	 61   GDISSLSLVNGTFSEIKDRMFSHLPSLQLLLLNSNSFTIIRDDAFAGLFH   110
CHIMPANZEE          61   GDISSLSLVNGTFSEIKDRMFSHLPSLQLLLLNSNSFTIIRDDAFAGLFH   110
DOG                 45   GDISSLSLVNGTFLEIKDRMFSHLPSLQLLLLNSNSFTVIRDDAFAGLFH   94
COW                205   GDISSLSLVNGTFSEIKDRMFSHLPSLQLLLLNSNSFTVIRDDAFAGLFH   254
MOUSE               58   GDISSLSLVNGTFLEIKDRMFSHLPSLQLLLLNSNSFTVIRDDAFAGLFH   107
RAT                248   GDISSLSLVNGTFLEIKDRMFSHLPSLQLLLLNSNSFTVIRDDAFAGLFH   297
CHICKEN             91   AGLGSLSLVNGTFSEVKDRMFSHLPSLQLLLLNSNSFTVIRDDAFAGLFH   140
ZEBRA FISH          52   NDVSSLSIVNGTFSEVKEAMFSHMPSLQLLLLNSNALTTVRDDAFSGLPH   101


HUMAN              111   LEYLFIEGNKIETISRNAFRGLRDLTHLSLANNHIKALPRDVFSDLDSLI   160
CHIMPANZEE         111   LEYLFIEGNKIETISRNAFRGLRDLTHLSLANNHIKALPRDVFSDLDSLI   160
DOG                 95   LEYLFIEGNKIETISRNAFRGLRDLTHLSLANNHIKALPRDVFSDLDSLI   144
COW                255   LEYLFIEGNKIETISRNAFRGLRDLTHLSLANNHIKALPRDVFSDLDSLI   304
MOUSE              108   LEYLFIEGNKIETISRNAFRGLRDLTHL----------------------   135
RAT                298   LEYLFIEGNKIETISRNAFRGLRDLTHLSLANNHIKALPRDVFSDLDSLI   347
CHICKEN            141   LEYLFIEGNKIETISRNAFRGLRDLTHLSLANNHLKTLPRDVFSDLDSLI   190
ZEBRA FISH         102   LEYLFIENNKIETTSKYSFRGLRDLTHLSLANNNIKALPRELFIDLDSLI   151


HUMAN              161   ELDLRGNKFECDCKAKWLYLWLKMTNSTVSDVLCIGPPEYQEKKLNDVTS   210
CHIMPANZEE         161   ELDLRGNKFECDCKAKWLYLWLKMTNSTVSDVLCIGPPEYQEKKLNDVTS   210
DOG                145   ELDLRGNKFECDCKAKWLYLWLKMTNSTVSDVLCIGPPEYQEKKLNDVPS   194
COW                305   ELDLRGNKFECDCKAKWLYLWLKMTNSTVSDVLCIGPPEYQEKKLNDVTS   354
MOUSE              136   --DLRGNKFECDCKAKWLYLWLKMTNSTVSDVLCIGPPEYQEKKLNEVTS   183
RAT                348   ELDLRGNKFECDCKAKWLYLWLKMTNSTVSDVLCIGPPEYQEKKLNDVTS   397
CHICKEN            191   ELDLRGNKFECDCKAKWLFLWLKMTNSTVSDVLCIGPAEYQDKKLNDVTS   240
ZEBRA FISH         152   ELDLRGNVFECDCRAKWLMMWLKSTNATVSDVLCAGPEEMKGKRLNDMAS   201


HUMAN              211   FDYECTTT--------------------------------DFVVHQTLPY   228
CHIMPANZEE         211   FDYECTTT--------------------------------DFVVHQTLPY   228
DOG                195   FDYECTTT--------------------------------DFVVHQTLPY   212
COW                355   FDYECTTT--------------------------------DFVVHQTLPY   372
MOUSE              184   FDYECTTTGPQTDEAKQRGWQLELSLGFCELIFVFQHPLSDFVVHQTLPY   233
RAT                398   FDYECTTT--------------------------------DFVVHQTLPY   415
CHICKEN            241   FDYECTTT--------------------------------DFVVHQILPY   258
ZEBRA FISH         202   LHNECIST--------------------------------DFIPLHSVPT   219


HUMAN              229   QSVSVDTFNSKNDVYVAIAQPSMENCMVLEWDHIEMNFRSYDNITGQSIV   278
CHIMPANZEE         229   QSVSVDTFNSKNDVYVAIAQPSMENCMVLEWDHIEMNFRSYDNITGQSIV   278
DOG                213   QSVSVDTFNSKNDVYVAIAQPSMENCMVLEWDHIEMNFRSYDNITGQSIV   262
COW                373   QSVSVDTFNSKNDVYVAIAQPSMENCMVLEWDHIEMNFRSYDNITGQSIV   422
MOUSE              234   QSVSVDTFNSKNDVYVAIAQPSMENCMVLEWDHIEMNFRSYDNITGQSIV   283
RAT                416   QSVSVDTFNSKNDVYVAIAQPSMENCMVLEWDHIEMNFRSYDNITGQSIV   465
CHICKEN            259   QSVSVDTFNSKNDVFVAIAQPSMENCMVLEWDHIEMNFRSYDNITGQSIV   308
ZEBRA FISH         220   ESLSVDTFSHKNDVYVAIAAPNAESCMVLQWDHIEMNFRTYDNITGQSIV   269


HUMAN              279   GCKAILIDDQVFVVVAQLFGGSHIYKYDESWTKFVKFQDIEVSRISKPND   328
CHIMPANZEE         279   GCKAILIDDQVFVVVAQLFGGSHIYKYDESWTKFVKFQDIEVSRISKPND   328
DOG                263   GCKAILIDDQVFVVVAQLFGGSHIYKYDESWTKFVKFQDIEVSRISKPND   312
COW                423   GCKAILIEDQVFVVVAQLFGGSHIYRYDESWTKFVKFQDIEVSRISKPND   472
MOUSE              284   GCKAILIDDQVFVVVAQLFGGSHIYKYDESWTKFVKFQDIEVSRISKPND   333
RAT                466   GCKAILIDDQVFVVVAQLFGGSHIYKYDESWTKFVKFQDIEVSRISKPND   515
CHICKEN            309   GCKAILVGDQVFVVVAQLFGGSHIYKYDESWTKFVKFQDIEVSRISKPND   358
ZEBRA FISH         270   GCKSVIIQNEVFVIVAQLFGGSHIYKFDEDQSKFSKFQDIEVSKISKPND   319


HUMAN              329   IELFQIDDETFFVIADSSKAGLSTVYKWNSKGFYSYQSLHEWFRDTDAEF   378
CHIMPANZEE         329   IELFQIDDETFFVIADSSKAGLSTVYKWNSKGFYSYQSLHEWFRDTDAEF   378
DOG                313   IELFQIEDETFFIIADSSKAGLSTVYKWNSKGFYSYQSLHEWFRDTDAEF   362
COW                473   IELFQIEDETFFVIADSSKAGLSTVYKWNSKGFYSYQSLHEWFRDTDAEF   522
MOUSE              334   IELFEIDDETFFIIADSSKAGLSTVYKWNSKGFYSYQSLHEWFRDTDAEF   383
RAT                516   IELFEIDDETFFIIADSSKAGLSTVYKWSSKGFYSYQSLHEWFRDTDAEF   565
CHICKEN            359   IELFEIDSEMFFVIADSSKAGLSTVYKWNNKGFYSYQSLHEWFRDTDAEF   408
ZEBRA FISH         320   IEAFQIGNDWFFIIADSSKAGLSTLYKWNDKGFYSYQSLHEWFRDTDAEF   369


HUMAN              379   VDIDGKSHLILSSRSQVPIILQWNKSSKKFVPHGDIPNMEDVLAVKSFRM   428
CHIMPANZEE         379   VDIDGKSHLILSSRSQVPIILQWNKSSKKFVPHGDIPNMEDVLAVKSFRM   428
DOG                363   VDIDGKSHLILSSRSQVPIILQWNKSSKKFVPHSDIPNMEDVLAVKSFRM   412
COW                523   VDIDGKSHLILSSRSQVPIILQWNKSSKKFVPHSDIPNMEDVLAVKSFRM   572
MOUSE              384   VDIDGKSHLILSSRSQVPIILQWNKSSKKFVPHGDIPNMEDVLAVKSFRM   433
RAT                566   VDIDGKSHLILSSRSQVPIILQWNKSSKKFVPHGDIPNMEDVLAVKSFRM   615
CHICKEN            409   LDIDGKSHLILSSRSQVPIILQWNKASKKFVPHGEIPNMEDVLAVKSFRM   458
ZEBRA FISH         370   VNLDGKAHLILASRSQVPVIYQWSRSTQKFTLQGEIPNMEDVVAVKAFWI   419


HUMAN              429   QNTLYLSLTRFIGDSRVMRWNSKQFVEIQALPSRGAMTLQPFSFKDNHYL   478
CHIMPANZEE         429   QNTLYLSLTRFIGDSRVMRWNSKQFVEIQALPSRGAMTLQPFSFKDNHYL   478
DOG                413   QNALYLSLTRFIGDSRVMRWNSKQFVEIQALPSRGAMTLQPFSFKDNHYL   462
COW                573   QNALYLSLTRFIGDSRVMRWNSKQFVEIQALPSRGAMTLQPFSFKENHYL   622
MOUSE              434   QNTLYLSLTRFIGDSRVMRWNSKQFVEVQALPSRGAMTLQPFSFKDNHYL   483
RAT                616   QNTLYLSLTRFIGDSRVMRWNSKQFVEVQALPSRGAMTLQPFSFKENHYL   665
CHICKEN            459   QNNLYITLTRFIGDSRVMKWNSKQFVEIQALPSRGAMTLQPFSFKNNYYL   508
ZEBRA FISH         420   KEDLYLAMTRYIGDSKVLHWTAKEFSEVQAIPSRGSMILQPFSFKERYYL   469


HUMAN              479   ALGSDYTFSQIYQWDKEKQLFKKFKEIYVQAPRSFTAVSTDRRDFFFASS   528
CHIMPANZEE         479   ALGSDYTFSQIYQWDKEKQLFKKFKEIYVQAPRSFTAVSTDRRDFFFASS   528
DOG                463   ALGSDYTFSQIYQWDKEKQLFKKFKEIYVQAPRCFTAVSTDRRDFFFASS   512
COW                623   ALGSDYTFSQIYQWDKEKQLFKKFKEIYVQAPRSFTAVSTDRRDFFFASS   672
MOUSE              484   ALGSDYTFSQIYQWDKEKQQFKKFKEIYVQAPRSFTAVSTDRRDFFFASS   533
RAT                666   ALGSDYTFSQIYQWDKEKQQFKKFKEIYVQAPRSFTAVSTDRRDFFFASS   715
CHICKEN            509   ALGSDYTFSQIYQWDGEKKLFRLFKEIYVQAPRSFTAVSTDRRDFFFASS   558
ZEBRA FISH         470   ALGSDYTFSQIYLWDAEKKVFERFKEVYIQAPRSFTVVSTDRRDFIFASS   519


HUMAN              529   FKGKTKIFEHIIVDLSL   545
CHIMPANZEE         529   FKGKTKIFEHIIVDLSL   545
DOG                513   FKGKTKIFEHIVVDLSL   529
COW                673   FKGKTKIFEHIVVDLSL   689
MOUSE              534   FKGKTKIFEHIIVDLSL   550
RAT                716   FKGKTKIFEHIIVDLSL   732
CHICKEN            559   FKGNTQIFEHVIIDLSL   575
ZEBRA FISH         520   FKGSTQIFEHIIIDLSL   536
