# Supplementary material for: LGI2 Truncation Causes a Remitting Focal Epilepsy in Dogs
Source: PLoS Genet. 2011 Jul 28;7(7):e1002194. doi: 10.1371/journal.pgen.1002194 (PMC3145619; doi:10.1371/journal.pgen.1002194)
Supplement: Table S1 — The coding regions and splice sites were screened for additional variants in the Lgi2 gene in two heterozygous affecteds (LAG001 and LAG099) and a set of adult-onset epileptic Lagottos or other puppies with juvenile epilepsy from other breeds including Barbets, Collies and German Shepherds. Although several variants were found none of them appear disease-causing. (DOC) [file pgen.1002194.s004.doc]

**Supporting information**

**Supporting Tables.**

**Table S1.** The coding regions and splice sites were screened for additional variants in the *Lgi2* gene in two heterozygous affecteds (LAG001 and LAG099) and a set of adult-onset epileptic *Lagottos* or other puppies with juvenile epilepsy from other breeds including *Barbets, Collies and German Shepherds*. Although several variants were found none of them appear disease-causing.

|  |  | Exon 1 | Intron 7 | Intron 7 | Exon 8 | Exon 8 | Exon 8 | Exon 8 | Exon 8 | Exon 8 | **Exon 8** |
| --- | --- | --- | --- | --- | --- | --- | --- | --- | --- | --- | --- |
|  |  | 35Gly>Gly | ― | ― | 327Ala>Ala | Ser>Ser | 392Phe>Phe | 457Lys>Asn | 466Ser>Cys | 505Arg>Ser | **518Lys>Stop** |
| Sample ID* | Description | 105C>T | 772+12C>T | 772+28T>G | 981T>C | 1107G>T | 1176C>T | 1371A>T | 1396A>T | 1515A>T | **1552A>T** |
| LAG005 | Epileptic puppy (litter 8) | C/C | C/C | T/T | T/T | G/T | C/C | A/A | A/A | A/A | **T/T** |
| LAG006 | Healthy puppy (litter 8) | ― | C/T | T/T | T/C | G/G | C/T | A/A | A/A | A/A | **A/T** |
| LAG068 | Epileptic puppy (litter 7) | C/C | C/C | T/T | T/T | G/G | C/C | A/A | A/A | A/A | **T/T** |
| LAG022 | Healthy puppy (litter 7) | C/C | C/T | T/T | T/C | G/G | C/T | A/A | A/A | A/A | **A/T** |
| LAG001 | Epileptic puppy (litter 9) | C/C | C/C | T/T | T/C | G/G | C/C | A/A | A/A | A/A | **A/T** |
| LAG041 | Healthy puppy (litter 9) | C/C | C/C | T/T | T/C | G/G | C/C | A/A | A/A | A/A | **A/T** |
| LAG099 | Epileptic puppy | C/C | C/T | T/T | T/C | G/G | C/T | A/A | A/A | A/A | **A/T** |
| LAG112 | Healthy puppy | C/C | C/T | T/T | T/C | G/G | C/T | A/A | A/A | A/A | **A/T** |
| LAG046 | Epileptic adult | C/C | T/T | T/T | C/C | G/G | T/T | A/A | A/A | A/A | **A/A** |
| LAG058 | Epileptic adult | C/C | T/T | T/T | C/C | G/G | T/T | A/A | A/A | A/A | **A/A** |
| LAG219 | Epileptic adult | C/C | T/T | T/T | C/C | G/G | T/T | A/A | A/A | A/A | **A/A** |
| LAG010 | Epileptic adult | T/T | C/C | T/T | T/T | G/G | C/C | A/A | A/A | A/A | **A/A** |
| LAG004 | Epileptic adult (litter 6) | C/C | C/C | T/T | T/T | G/G | C/C | A/A | A/A | A/A | **T/T** |
| LAG063 | Epileptic puppy (litter 6) | C/C | C/C | T/T | T/T | G/G | C/C | A/A | A/A | A/A | **T/T** |
| BAR010 | epileptic puppy | C/C | C/C | T/G | T/C | G/G | C/T | A/A | A/A | A/A | **A/A** |
| BAR011 | epileptic puppy | ― | C/C | T/G | T/T | G/G | C/C | A/A | A/A | A/A | **A/A** |
| BAR013 | epileptic puppy | C/C | ― | ― | T/T | G/G | C/C | A/A | A/A | A/A | **A/A** |
| BAR005 | healthy | C/C | C/C | T/T | T/T | G/G | C/C | A/T | A/T | A/T | **A/A** |
| BAR015 | healthy | C/C | C/C | G/G | C/C | G/G | T/T | A/A | A/A | A/A | **A/A** |
| CLK038 | epileptic puppy | C/C | C/C | T/T | T/C | G/G | C/C | A/A | A/A | A/A | **A/A** |
| CLK039 | epileptic puppy | C/C | C/C | T/T | T/C | ― | ― | A/A | A/A | A/A | **A/A** |
| CLK040 | epileptic puppy | C/C | C/C | T/T | T/C | G/G | C/C | A/A | A/A | A/A | **A/A** |
| SP390 | epileptic puppy | C/C | C/C | T/T | T/T | G/G | C/C | A/A | A/A | A/A | **A/A** |

* LAG samples are from *Lagotto Romagnolos*, BAR from *Barbets*, CLK from *Collies* and SP is from *German Shepherd.*
