# Supplementary material for: LGI2 Truncation Causes a Remitting Focal Epilepsy in Dogs
Source: PLoS Genet. 2011 Jul 28;7(7):e1002194. doi: 10.1371/journal.pgen.1002194 (PMC3145619; doi:10.1371/journal.pgen.1002194)
Supplement: Table S2 — Genotype frequencies of the Lgi2 c.1552A>T (p.K518X) mutation tested from Lagotto Romagnolo dogs in different countries. (DOC) [file pgen.1002194.s005.doc]

**Table S2.** Genotype frequencies of the *Lgi2* c.1552A>T (p.K518X) mutation tested from *Lagotto Romagnolo* dogs in different countries.

| **Country** | **n A/A** | **% A/A** | **n A/T** | **% A/T** | **n T/T** | **%T/T** | **Total** |
| --- | --- | --- | --- | --- | --- | --- | --- |
| Finland | 209 | 59% | 112 | 32% | 34 | 10% | 355 |
| Switzerland | 99 | 61% | 52 | 32% | 10 | 6% | 161 |
| Sweden | 37 | 62% | 19 | 32% | 4 | 7% | 60 |
| **Total** | **345** | **60%** | **183** | **32%** | **48** | **8%** | **576** |
