# Supplementary material for: LGI2 Truncation Causes a Remitting Focal Epilepsy in Dogs
Source: PLoS Genet. 2011 Jul 28;7(7):e1002194. doi: 10.1371/journal.pgen.1002194 (PMC3145619; doi:10.1371/journal.pgen.1002194)
Supplement: Table S3 — The Lgi2 c.1552A>T mutation is breed-specific. Mutation was screened altogether from 114 adult-onset epilepsy cases and eight juvenile epilepsy cases that represented 40 different breeds. None of the studied dogs carried the mutant allele present in Lagottos. (DOC) [file pgen.1002194.s006.doc]

**Table S3.** The *Lgi2* c.1552A>T mutation is breed-specific. Mutation was screened altogether from 114 adult-onset epilepsy cases and eight juvenile epilepsy cases that represented 40 different breeds. None of the studied dogs carried the mutant allele present in *Lagottos*.

| **Breed** | **Adult onset epilepsy** | **Juvenile epilepsy** |
| --- | --- | --- |
| Australian Shepherd | 3 | 0 |
| Barbet | 0 | 3 |
| Basenji | 3 | 0 |
| Basset griffon vendeen | 3 | 0 |
| Beagle | 3 | 0 |
| Belgian Shepherd | 3 | 0 |
| Bernise Mountain Dog | 3 | 0 |
| Border Collie | 3 | 0 |
| Border Terrier | 3 | 1 |
| Chinese Crested Dog | 3 | 0 |
| Collie, smooth | 0 | 3 |
| Curly Coated Retriever | 3 | 0 |
| Dachshund, Long-haired | 3 | 0 |
| Dalmatian | 3 | 0 |
| Finnish Hound | 3 | 0 |
| Finnish Lapphund | 3 | 0 |
| Finnish Spitz | 3 | 0 |
| Flat Coated Retriever | 3 | 0 |
| German Pointing Dog, wire-haired | 3 | 0 |
| German Shepherd | 3 | 1 |
| Golden Retriever | 3 | 0 |
| Irish Water Spaniel | 3 | 0 |
| Japanese Spitz | 3 | 0 |
| Keeshond | 3 | 0 |
| Kromfohrländer | 3 | 0 |
| Labrador Retriever | 3 | 0 |
| Lapponian Herder | 3 | 0 |
| Miniature Pincher | 3 | 0 |
| Miniature Schnauzer | 3 | 0 |
| Norwich Terrier | 3 | 0 |
| Nova Scotia Duck Tolling Retriever | 3 | 0 |
| Peruvian Hairless Dog | 3 | 0 |
| Pug | 3 | 0 |
| Pyrenean Shepherd | 3 | 0 |
| Rottweiler | 3 | 0 |
| Saluki | 3 | 0 |
| Schipperke | 3 | 0 |
| Siperian Husky | 3 | 0 |
| Toy Poodle | 3 | 0 |
| Whippet | 3 | 0 |
| Total | 114 | 8 |
