# Supplementary material for: LGI2 Truncation Causes a Remitting Focal Epilepsy in Dogs
Source: PLoS Genet. 2011 Jul 28;7(7):e1002194. doi: 10.1371/journal.pgen.1002194 (PMC3145619; doi:10.1371/journal.pgen.1002194)
Supplement: Table S4 — Primers used in the study. (DOC) [file pgen.1002194.s007.doc]

**Table S4.** Primers used in the study

| ***Forward primer*** | ***Sequence (5'->3')*** | ***Reverse primer*** | ***Sequence (5'->3')*** | ***Tm*** | ***Product size*** |
| --- | --- | --- | --- | --- | --- |
| Lgi2ex1F | gattccgagCCTCGTGCG | Lgi2ex1R | caggcactcacAGGGAGC | 57 | 152 |
| Lgi2ex2F | cccatcctgcaatcacttct | Lgi2ex2R | gaaggagtgacgccaaagag | 57 | 245 |
| Lgi2ex3F | tgtacatcactgcgctgaaa | Lgi2ex3R | cctaagagatgccgttcctg | 57 | 200 |
| Lgi2ex4F | ggttttgttccgagtatcgtg | Lgi2ex4R | agggaccaggaagtggatct | 57 | 227 |
| Lgi2ex5F | gagtgacagggcatgaggat | Lgi2ex5R | gattaactggagcccaacga | 62 | 180 |
| Lgi2ex6F | ggcatatctgtttctccgtct | Lgi2ex6R | ccattccctgtggatgttct | 57 | 292 |
| Lgi2ex7F | cgaagggaagcaggttactg | Lgi2ex7R | ttgaggccacaatgaaatga | 57 | 298 |
| Lgi2ex8aF | cattcttacctaatcccctctcg | Lgi2ex8aR | gcctttgctgttccatttatacac | 57 | 300 |
| Lgi2ex8bF | atcgctgacagctccaaag | Lgi2ex8bR | aaagggctgcagggtcat | 62 | 387 |
| Lgi2ex8cF | gggtcatgaggtggaacagt | Lgi2ex8cR | ttggctttccatttgcttct | 57 | 369 |
| **Exon-specific primers for RNA sequencing** | |  |  |  |  |
| Lgi2_fromEx4_toEx6_F | ctccgtgacctgactcacct | Lgi2_fromEx4_toEx6_R | atactctggtggaccgatgc | 57 | 207 |
